# Closing Remarks for the Special Issue “Advances in Endovascular Therapies and Acute Stroke Management”

**DOI:** 10.3390/life16081282

**Published:** 2026-08-03

**Authors:** Anna Podlasek

**Affiliations:** 1 Image Guided Therapy Research Facility, University of Dundee, Dundee DD1 4HN, UK; apodlasek001@dundee.ac.uk; 2 School of Medicine, University of St Andrews, St Andrews KY16 9ST, UK

**Keywords:** stroke, mechanical thrombectomy, brain imaging, thrombolysis, neuroprotection, acute kidney injury

Stroke remains one of the leading causes of death and acquired disability worldwide [1]. Acute ischemic stroke due to large vessel occlusion (LVO) is responsible for a disproportionate share of long-term disability, and is also the subgroup in which mechanical thrombectomy (MT) combined with intravenous thrombolysis (IVT) has produced some of the most striking treatment effects in modern neurology [2,3,4,5,6,7]. A decade after the landmark thrombectomy trials, the questions facing the field have shifted from “whether to treat” to whom, when, where, and how [8]. The five contributions gathered in this Special Issue follow a single patient through that journey, from the front door of the hospital to long-term functional recovery, and together highlight both where progress has been most rapid and where genuine uncertainty remains.

The journey begins with imaging and patient selection. Bobe et al. [9] provide a systematic review of CT perfusion (CTP) in acute ischemic stroke, synthesising 39 studies across eight thematic domains: core and penumbra estimation, prognosis, treatment selection, collateral assessment, software validation, technical parameters, reliability, and safety. CTP undeniably enriches our understanding of tissue viability and can be helpful when patient selection is genuinely uncertain. However, the review also makes clear that CTP is not a panacea and, in everyday practice, is often not strictly necessary. For the great majority of LVO patients presenting in the conventional early window, non-contrast CT and CT angiography alone provide adequate selection without the additional contrast load, radiation, or workflow time required by perfusion imaging. Even in the extended window, the supporting evidence is overwhelmingly observational: registry data such as those reported by Dhillon et al. [10] are consistent with improved functional outcomes when perfusion-based selection is used between 6 and 24 h, yet such indirect comparisons are vulnerable to selection bias and to differences in baseline patient characteristics, and should therefore be interpreted with caution while awaiting confirmatory data from prospective randomised trials. The technical limitations highlighted by Bobe et al. [9] reinforce this point. Tissue thresholds vary across vendors; the well-described “ghost infarct core” phenomenon can lead to systematic overestimation of irreversible injury, particularly in the early hours; meaningful differences exist between post-processing software packages, with the same raw data yielding non-trivially different core volumes; and diffusion-weighted MRI remains the more accurate reference for ischemic core estimation. Until standardisation, software validation, and randomised evidence catch up, perfusion imaging is best understood as a selective adjunct for borderline cases rather than a default in every workflow.

Once a patient has been selected for treatment, the next determinant of outcome is whether they can reach a thrombectomy-capable team in time. Dziadkiewicz et al. [11] address this systems-of-care question through a five-year experience of a thrombectomy-capable stroke centre (TCSC) in Northern Poland [12]. Comparing the first 100 patients with the subsequent 150, the authors capture the simultaneous evolution of guidelines, the establishment of 24/7 service, and the transition from a “direct-admission-only” model to a mixed model that includes drip-and-ship referrals from primary stroke centres. Despite longer in-hospital workflows associated with the drip-and-ship pathway and the broader inclusion of extended-window patients, clinical outcomes and complication rates remained favourable when compared with regional and national benchmarks. The data illustrate two replicable lessons: the impact of the operator and team learning curve on procedural metrics, and the feasibility of lower-volume TCSCs in widening access to mechanical thrombectomy in regions where comprehensive stroke centres are geographically distant, a message of clear relevance well beyond Poland.

Procedural safety is the next stop on the patient journey, and lessons from adjacent vascular territories continue to inform neurovascular practice. Wittig et al. [13] report on acute kidney injury (AKI) following peripheral interventions performed primarily with carbon dioxide (CO_2_) angiography in 340 patients with peripheral artery disease and chronic kidney disease. Postinterventional AKI occurred in 13.2% of patients, the majority with KDIGO stage 1 injury. Crucially, multivariate analysis identified hypertension, congestive heart failure, and coronary artery disease—rather than the volume of bailout iodinated contrast—as the dominant independent risk factors. For neurointerventionalists treating older, multimorbid stroke patients, the implication is that AKI risk is driven primarily by the cardiovascular and renal substrate rather than by contrast volume alone, reinforcing the case for individualised periprocedural risk assessment, careful haemodynamic management, and judicious medication review.

Even after reperfusion has been achieved, outcome remains heterogeneous, and prognostic models still rely largely on cohorts dominated by anterior circulation strokes. Bolognese et al. [14] address this gap directly, showing in 410 patients that the predictors of poor three-month functional outcome differ meaningfully between infratentorial and supratentorial ischemic stroke. In the infratentorial group, atrial fibrillation, reduced estimated glomerular filtration rate, and (marginally) diabetes mellitus emerged as the dominant predictors, whereas in supratentorial stroke the NIHSS at admission was the single strongest determinant, accompanied by age. The authors also highlight the prognostic relevance of high-sensitivity cardiac troponin T, particularly in posterior circulation events, suggesting that subgroup-specific prognostic models are likely to outperform one-size-fits-all approaches.

Finally, the persistent gap between high recanalisation rates and modest improvements in patient-reported function continues to motivate the search for adjunctive neuroprotective strategies. Filimonov et al. [15] approach this question through two-sample Mendelian randomisation, using thirteen single-nucleotide polymorphisms predicting free triiodothyronine (fT3) levels to interrogate functional outcome at three months. A genetically predicted increase in fT3 was associated with a meaningful reduction in the odds of an unfavourable outcome (OR 0.581, 95% CI 0.37–0.92, *p* = 0.018), with consistent results from sensitivity analyses. Although the analysis is restricted to individuals of European ancestry and the underlying mechanism remains to be elucidated, the study adds a genetically informed signal to a growing body of clinical data and strengthens the rationale for prospective trials of thyroid hormone analogues as candidate neuroprotectants.

Read together, these five papers trace a single arc—from the emergency department, through imaging, transfer, procedure, and prognostication, to recovery—and three cross-cutting themes emerge. The first is precision: better imaging, better biomarkers, and better stratification are converging to support more individualised decisions [16], while also exposing the limits of any single modality. The second is equity of access: every gain in trial-derived efficacy is only as valuable as the proportion of eligible patients who can reach a capable team in time, which makes the development of thrombectomy-capable centres, transfer networks, and operator training programmes a clinical and ethical priority. The third is humility about evidence: much of what currently informs extended-window selection, neuroprotection, and biomarker-based stratification rests on observational data, and indirect comparisons must continue to be interpreted with caution while we await definitive randomised trials.

As Guest Editor, I am grateful to the authors who contributed their work to this Special Issue, to the many anonymous peer reviewers whose careful comments shaped each manuscript, and to the editorial staff at *Life* for their professionalism throughout. It has been a privilege to work with such a committed community.
